# Identification of a six‐microRNA signature as a potential diagnostic biomarker in breast cancer tissues

**DOI:** 10.1002/jcla.24010

**Published:** 2021-09-15

**Authors:** Mojdeh Mahmoudian, Ehsan Razmara, Bashdar Mahmud Hussen, Mandana Simiyari, Nazanin Lotfizadeh, Hoda Motaghed, Arefeh Khazraei Monfared, Maryam Montazeri, Sadegh Babashah

**Affiliations:** ^1^ Department of Genetics Faculty of Sciences Science and Research Branch Islamic Azad University Tehran Iran; ^2^ Department of Medical Genetics Faculty of Medical Sciences Tarbiat Modares University Tehran Iran; ^3^ Department of Pharmacognosy College of Pharmacy Hawler Medical University Kurdistan Region Iraq; ^4^ Department of Veterinary Medicine Faculty of Veterinary Medicine Tabriz Branch Islamic Azad University Tabriz Iran; ^5^ Department of Biology Faculty of Advanced Science and Technology Tehran Medical Sciences Islamic Azad University Tehran Iran; ^6^ Department of Biology Faculty of Biological Sciences Islamic Azad University‐Tehran North Branch Tehran Iran; ^7^ Department of Medical Biotechnology Faculty of Advanced Science and Technology Tehran Medical Sciences Islamic Azad University Tehran Iran; ^8^ Department of Molecular Genetics Faculty of Biological Sciences Tarbiat Modares University Tehran Iran

**Keywords:** biomarker, breast cancer, breast tissue, diagnosis, miRNA signature

## Abstract

**Background:**

Breast cancer (BC) is by far the most common malignancy among women. Epigenetic modulators, microRNAs in particular, may set stages for BC development and its progression. Herein, we aimed to assess the diagnostic potentiality of a signature of six miRNAs (i.e., hsa‐miR‐25‐3p, ‐29a‐5p, ‐105‐3p, ‐181b1‐5p, ‐335‐5p, and ‐339‐5p) in BC and adjacent non‐tumor tissues.

**Methods:**

A pair of 50 tumor and adjacent non‐tumor samples were taken from BC patients. The expression of each candidate miRNA was measured using quantitative reverse transcription PCR. To investigate the possible roles of each miRNA and their impressions on BC prognosis, *in silico* tools were used. Receiver operating characteristic (ROC) curves were performed to determine the diagnostic accuracy of each miRNA and the possible association of their expression with clinicopathological characteristics was analyzed.

**Results:**

Our findings showed the upregulation of hsa‐miR‐25‐3p, ‐29a‐5p, ‐105‐3p, and ‐181b1‐5p, and the downregulation of hsa‐miR‐335‐5p and ‐339‐5p in BC tumor compared to corresponding adjacent tissues. Except for hsa‐miR‐339‐5p, the up‐/down‐regulation of the candidate miRNAs was associated with TNM stages. Except for hsa‐miR‐105‐3p, each candidate miRNA was correlated with HER‐2 status. ROC curve analysis showed that the signature of six‐miRNA is a potential biomarker distinguishing between tumor and non‐tumor breast tissue samples.

**Conclusion:**

We showed that the dysregulation of a novel signature of six‐miRNA can be used as a potential biomarker for BC diagnosis.

## Graphical abstract

Our findings showed the upregulation of hsa‐miR‐25‐3p, ‐29a‐5p, ‐105‐3p, and ‐181b1‐5p in BC tumor compared to its corresponding adjacent tissues. Downregulation of hsa‐miR‐335‐5p and ‐339‐5p was evident in BC tumor compared to its corresponding adjacent tissues. Except for hsa‐miR‐105‐3p, each candidate miRNA was correlated with HER‐2 status. The signature of six miRNAs is a potential biomarker to distinguish between tumor and non‐tumor breast tissue samples.

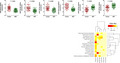

## 1 INTRODUCTION

Breast cancer (BC) is responsible for about 25.2% of all women cancers worldwide, suggesting an increasingly rising trend.^1^ BC develops as localized disease, but it can metastasize to distant organs (e.g., bone, lung, liver, and brain) and pose the patients' life danger.^2^ Accordingly, patients whose disease is diagnosed late often have a low rate of prognosis. Needless to say that if BC is diagnosed as early as possible, it improves the patients' life.^3^, ^4^

Among different imaging techniques that are used for BC diagnosis, mammography is still a gold standard technique.^5^ This method, on the other hand, is problematic due to false‐negative and/or ‐positive diagnoses, required biopsy, low sensitivity, and imposing psychological stresses.^6^, ^7^, ^8^ Hence, introducing noninvasive methods is necessary that can discriminate tumor and healthy markers as early as possible with good enough sensitivity. Different attempts have been pushed back the frontiers of knowledge so far to develop diagnostic and therapeutic resources for BC patients; for instance, it has been recently identified that non‐coding RNAs (e.g., miRNAs) can be used as a biomarker to diagnose the early stages of cancer and also follow‐up the patients to determine treatment efficacy.^9^, ^10^, ^11^, ^12^

As a class of small non‐coding RNAs that are composed of <22 nucleotides, microRNAs (miRNAs and miRs) regulate gene expression at the post‐transcriptional level.^12^, ^13^, ^14^ These molecules play in a variety of biological processes such as cell proliferation, differentiation, and apoptosis,^12^, ^14^ and their dysregulated expression—i.e., up‐regulation or down‐regulation,^9^ has been identified in different tumors. According to their functions, miRNAs are classified into oncogenes or tumor suppressors.^15^, ^16^ It has been also suggested that miRNA expression profiling is of importance because it paves the way toward using these molecules to determine the diagnosis, staging, prognosis, and response to treatment in BC.^10^

In this study, we aimed to examine whether a signature of six‐miRNA (i.e., hsa‐miR‐25‐3p, ‐29a‐5p, 105‐3p, ‐181b1‐5p, ‐335‐5p, and ‐339‐5p) can be used as a biomarker to distinguish BC from adjacent non‐tumor tissues (ANT). We selected the candidate miRNAs based on literature reviews and data mining for those that were actively involved in BC pathogenesis. Moreover, we looked into the possible relationships between the expression of these miRNAs and patients' clinicopathological features and *HER‐2* expression status. To assess which miRNA discriminates BC from ANT tissues, ROC curve analysis was used. Besides, to show targets and evaluate the prognostic value of each candidate miRNA, in *silico* analysis was performed.

## 2 MATERIAL AND METHODS

### 2.1 Patients

Formalin‐fixed paraffin‐embedded (FFPE) samples were obtained from BC patients who were admitted by Imam Khomeini Cancer Institute, Tehran, Iran. The study protocol was approved by the Ethics Committee of Islamic Azad University, Tehran, Iran, and before doing the experiment, each participant voluntarily gave their written informed consent. They also signed an informed consent form to collect their tissue samples. Breast tissues were collected using standard operating procedures that had been undertaken at National Cancer Center Hospital. In total, a pair of 50 tumor and ANT samples were collected from the patients and were assessed histopathologically based on the World Health Organization (WHO) criteria for the histologic grade,^17^ the TNM system for stage classification, and human epidermal growth factor receptor 2 (HER‐2) status. Some important clinicopathological features for these tissue samples such as tumor stage, estrogen receptor, and HER‐2 status are summarized in Table 1.

**TABLE 1:** The clinicopathological characteristics of breast cancer patients

Characteristics	No. (%)
Age (years)	
<50	18 (36)
≥50	32 (64)
Tumor size (cm)	
<2	12 (24)
≥2	38 (76)
Primary tumor (T stage) (cm)	
T1: ≤2	14 (28)
T2: >2–≤5	27 (54)
T3: >5	9 (18)
Regional lymph nodes (N stage)	
NX	4 (8)
N0	14 (28)
N1	12 (24)
N2	14 (28)
N3	6 (12)
Distant metastasis (M stage)	
MX	3 (6)
M0	17 (34)
M1	30 (60)
Tumor stage	
I + II	29 (58)
III	21 (42)
Estrogen receptor status	
Negative	17 (34)
Positive	30 (60)
Unknown	3 (6)
Progesterone receptor status	
Negative	20 (40)
Positive	28 (56)
Unknown	2 (4)
Human epidermal growth factor receptor 2 (HER‐2) status	
Negative	26 (52)
Positive	24 (48)

### 2.2 RNA isolation and quality evaluation

Formalin‐fixed paraffin‐embedded blocks were cut, mounted on slides, and tumor tissue was scraped into 1.5‐ml tubes by needle macrodissection for subsequent RNA extraction. Briefly, 1 ml of xylene was added into the 4 pieces of 20‐µm‐thick FFPE sections to remove traces of paraffin. The tissues were digested with protease K at 50°C overnight. Total RNA was extracted from FFPE tissues using TRIzol reagents (Invitrogen, CA, USA). Before extraction, the samples were washed several times using xylene to solubilize and remove the paraffin. The concentration of RNA samples was determined using the NanoDrop 2000c (Thermo Fisher Scientific), while the integrity was confirmed using 2% gel electrophoresis. To eliminate any remaining DNA contaminations, the samples were treated with RNase‐free DNase (Ambion, Austin, TX, USA).

### 2.3 Choosing candidate miRNA for experimental validation

We selected the candidate miRNAs based on literature reviews and data mining for those that were actively involved in BC pathogenesis. The candidate miRNAs were dysregulated and detectable in human BC. Besides, each of the candidate miRNA must have been annotated straightforwardly in miRBase 22.1. Finally, we selected candidate miRNAs that might have been made a contribution to different signaling pathways in BC pathogenesis. Considering these criteria, hsa‐miR‐25‐3p, ‐29a‐5p, 105‐3p, ‐181b1‐5p, ‐335‐5p, and ‐339‐5p were nominated to have their expression analyzed in BC and ANT tissue samples.

### 2.4 Complementary DNA (cDNA) synthesis and RT‐qPCR

Poly‐(A)‐tailing and cDNA synthesis were carried out by reverse transcription of approximately 1 μg of total RNA using MiR‐Amp Kit (ParsGenome, Tehran, Iran). The anchored oligo(dT) sequence for cDNA synthesis was as follows: GCGTCGACTAGTACAACTCAAGGTTCTTCCAGTCACGACGTTTTTTTTTTTTTTTTTT(N).

The expression of each miRNA was assessed by miRNA‐specific primer and RT‐qPCR master mix kit (ParsGenome, Tehran, Iran). The RT‐qPCR was performed on an ABI StepOne Sequence Detection System (Applied Biosystems, CA, USA) under the following conditions: initial denaturation stage at 95°C for 5 min, 40 cycles of denaturation at 95°C for 10 s, and annealing/extension stage at 60°C for 30 s. To determine the specificity of PCR products, melt curve analysis was performed after accomplishing the proliferation that was included 95°C for 15 s, 60°C for 30 s, and 90°C for 15 s.

U48 snRNA (*SNORD48*) was used to normalize the relative expression of each candidate miRNA. The calculation was performed using the 2^−ΔΔCt^ method where ΔCt = Ct (Target)‐Ct (Reference) and fold changes were calculated using this method. The primer sequences were as follows: *U48*‐Forward: 5′‐TGACCCCAGGTAACTCTGAGTGTGT‐3′ Universal‐Reverse: 5′‐GCGTCGACTAGTACAACTCAAG‐3′, hsa‐miR‐25‐3p: 5′‐CAUUGCACUUGUCUCGGUCUGA‐3′; hsa‐miR‐29a‐5p: 5′‐UAGCACCAUCUGAAAUCGGUUA‐3′; hsa‐miR‐105‐3p: 5′‐ACGGAUGUUUGAGCAUGUGCUA‐3′; hsa‐miR‐181b1‐5p: 5′‐AACAUUCAUUGCUGUCGGUGGGU‐3′; hsa‐miR‐181b1‐5p: 5′‐AACAUUCAUUGCUGUCGGUGGGU‐3′; hsa‐miR‐335‐5p: 5′‐UCAAGAGCAAUAACGAAAAAUGU‐3′; and hsa‐miR‐339‐5p: 5′‐UCCCUGUCCUCCAGGAGCUCACG‐3′.

### 2.5 Functional enrichment analysis

To assess the target potential biological processes and pathways of each candidate miRNA, bioinformatics analyses were undertaken using the Gene Ontology (GO) biological process and Kyoto Encyclopedia of Genes and Genomes (KEGG) pathway options of the DIANA‐miRPath v3.0.^18^ In the context of interactive interaction networks, miRTargetLink Human was used which provided comprehensive information on human miRNA‐mRNA interactions.^19^ Furthermore, the miRWalk 2.0 (http://zmf.umm.uni‐heidelberg.de/apps/zmf/mirwalk3/index.html) was used to predict miRNA target genes.^20^ This platform integrates data from at least 12 miRNA‐target databases, containing PITA, miRNAMap, RNA22, miRanda, MiRWalk, MicroT4prediction datasets, miRBridge, miRDB, miRMap, PICTAR2, RNAhybrid, and Targetscan. We only just focused on the 3́‐untranslated regions as the primary base‐pairing regions. We considered the miRNA‐gene pairs that were in common in at least 5 databases (*p*‐values <0.05).

### 2.6 Identification of candidate prognostic markers using Kaplan–Meier method

Prognosis is a critical parameter determining medication efficacy and the relationship between gene expression and disease progression.^21^ Since the BC patients' following up was impossible, we used the *Kaplan–Meier plotter database* ^22^ and *Pan‐Cancer Tool* ^23^ to assess the possible relationship between the expression of each miRNA and the prognosis. This database involves the genome‐wide gene expression profiles of more than 5,000 BC samples. We divided the samples into two classes based on the upper quartile expression value of each miRNA, and the log‐rank test was used to determine differences in ‘overall survival’ between the high and low expression groups. Hazard ratio, 95% confidence intervals (CI), and log‐rank *p*‐values were calculated.

### 2.7 Statistical analysis

We used SPSS v.26.0 (IBM Corp., Armonk, NY, USA) and GraphPad Prism v.8.0 (GraphPad Software, Inc., La Jolla, CA, USA). The S*tudent's t‐test* was used and *p*‐values <0.05 were considered statistically significant. All experiments were repeated at least three times. The receiver operating characteristic (ROC) curves were used to determine the diagnostic value of the signature, particularly by calculating the area under the curve (AUC) with at least 95% of confidence intervals (CI).

## 3 RESULTS

### 3.1 Expression analysis of the signature in tumor and ANT tissue samples

Using *in silico* tools and literature reviews, six miRNAs were selected and their expression patterns and biomarker potentialities were further analyzed. These miRNAs could make a contribution to the progression of BC.

To measure the reaction efficiency for each primer, five‐fold serial dilutions of cDNA samples were prepared. Standard curves via plotting the logarithmic amount of serially diluted cDNA input against the corresponding Ct values were exploited. The efficiency of RT‐qPCR was calculated according to the slope of the standard curve and the following equation: E = 10^(−1/slope)^. As shown in Table S1, the amplification efficiency of each miRNA and internal control was roughly equal with a high linear correlation, showing the validity of the assay. Moreover, dissociation curve analysis was used to endorse the uniqueness and specificity of the amplified products. Single and sharp peaks of the melting curves showed no primer‐dimer or non‐specific products (Figure S1).

**FIGURE 1:**
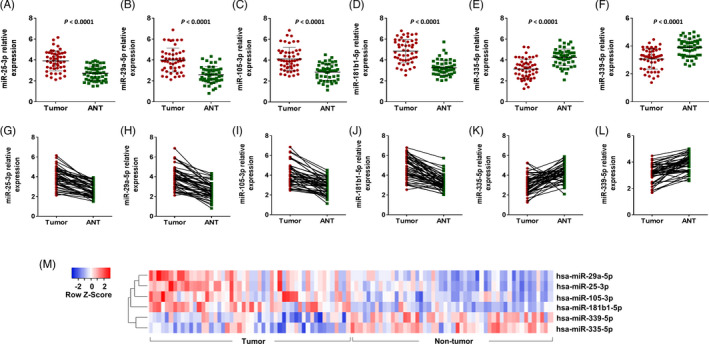
The candidate miRNA expression in breast cancer (BC) tumor and adjacent non‐tumor (ANT) tissues. The upregulation of hsa‐miR‐25‐3p (A), hsa‐miR‐29a‐5p (B), hsa‐miR‐105‐3p (C), and hsa‐miR‐181b1‐5p (D) was detected in tumor tissues compared to the adjacent non‐tumor samples. On the other hand, the downregulation of hsa‐miR‐335‐5p (E) and hsa‐miR‐339‐5p (F) was identified in tumor tissues. (G–L) Before–after bars showed the downregulation and upregulation of the putative miRNAs. (M) Unsupervised hierarchical clustering analysis underscored the differentially expressed miRNAs in tumor and non‐tumor BC tissues. The heatmap (Euclidian distance, complete linkage) represents miRNAs with high expression in red and low expression in blue. The heatmap was depicted using Heatmapper (http://www.heatmapper.ca/expression/)

RT‐qPCR was used to evaluate the expression of each candidate miRNA in 50 pairs of BC and ANT tissue samples. Our results showed the upregulation of hsa‐miR‐25‐3p, ‐29a‐5p, 105‐3p, and 181b1‐5p (*p*‐values <0.0001) in tumor samples compared to ANT tissue samples (Figure 1A–L). Furthermore, a significant downregulation of hsa‐miR‐335‐5p and hsa‐miR‐339‐5p (*p*‐values <0.0001) was detected in BC tumor tissues (Figure 1A–L). Unsupervised hierarchical clustering analysis of the expression of the candidate miRNAs showed that this set of a six‐marker signature can consummately discriminate BC tumor and ANT tissues (Figure 1M).

### 3.2 Correlation between miRNA expression levels and the level of malignancy and HER‐2 status

**FIGURE 2:**
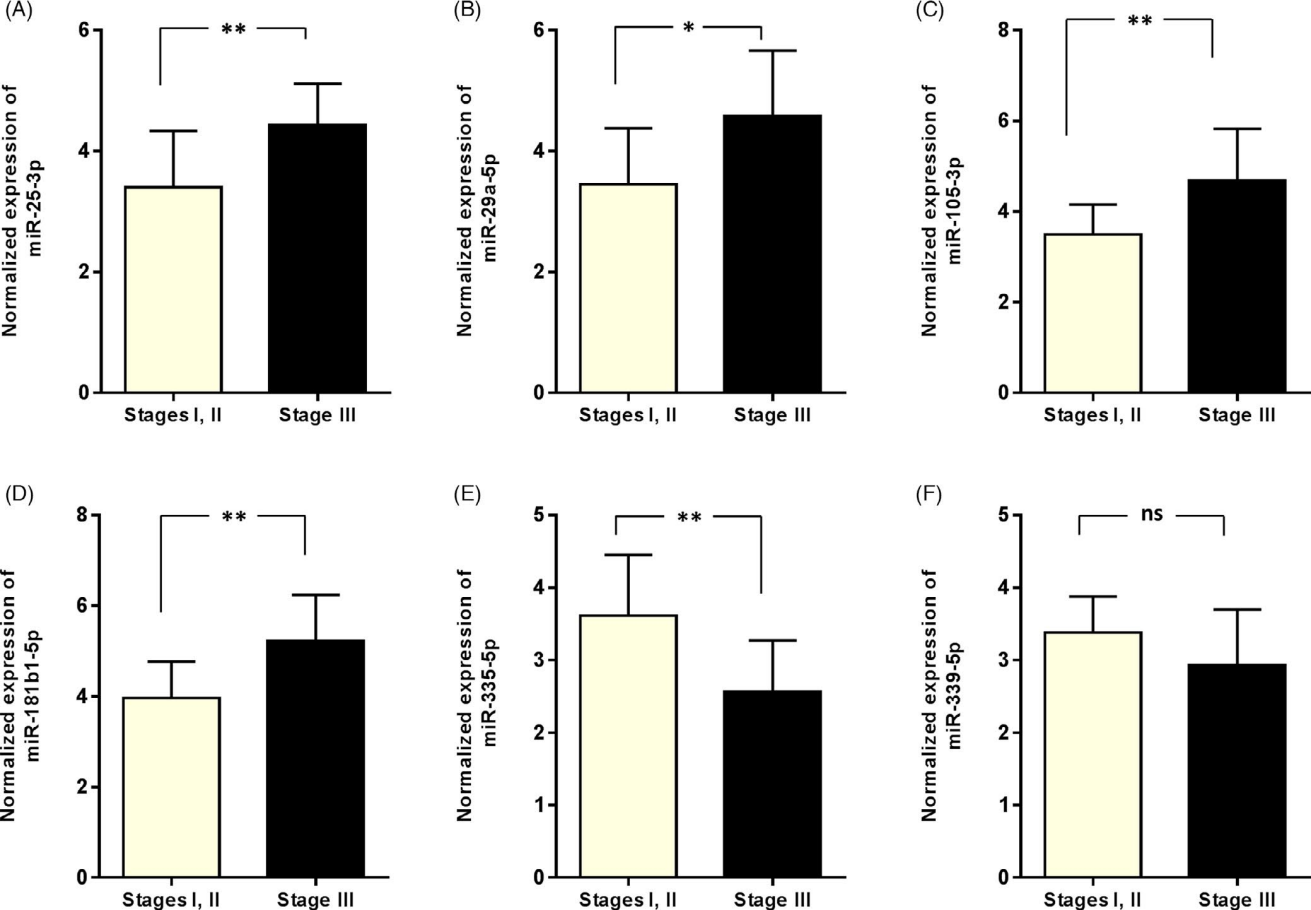
miRNA expression levels in breast cancer patients with different tumor stages. A reverse association was identified between the upregulation of hsa‐miR‐25‐3p (A), hsa‐miR‐29a‐5p (B), hsa‐miR‐105‐3p (C), and hsa‐miR‐181b1‐5p (D) with advanced tumor stages. On the other side, the downregulation of hsa‐miR‐335‐5p was mostly observed in advanced stages (**p‐value <0.01). No significant correlation was observed between the expression of hsa‐miR‐339‐5p and tumor stages. In this figure: *p‐value <0.05, **p‐value <0.01

We also assessed the possible association between the signature expression and clinicopathological characteristics of the BC patients. We pointed out that the expression of hsa‐miR‐25‐3p, ‐29a‐5p, 105‐3p, and 181b1‐5p was decreased in early stages (i.e., stages I and II) of BC tumorigenesis, while the expression of hsa‐miR‐335‐5p increased in the stages. Similarly, the expression of hsa‐miR‐339‐5p was insignificantly increased in the early stages of BC (Figure 2A–F).

**FIGURE 3:**
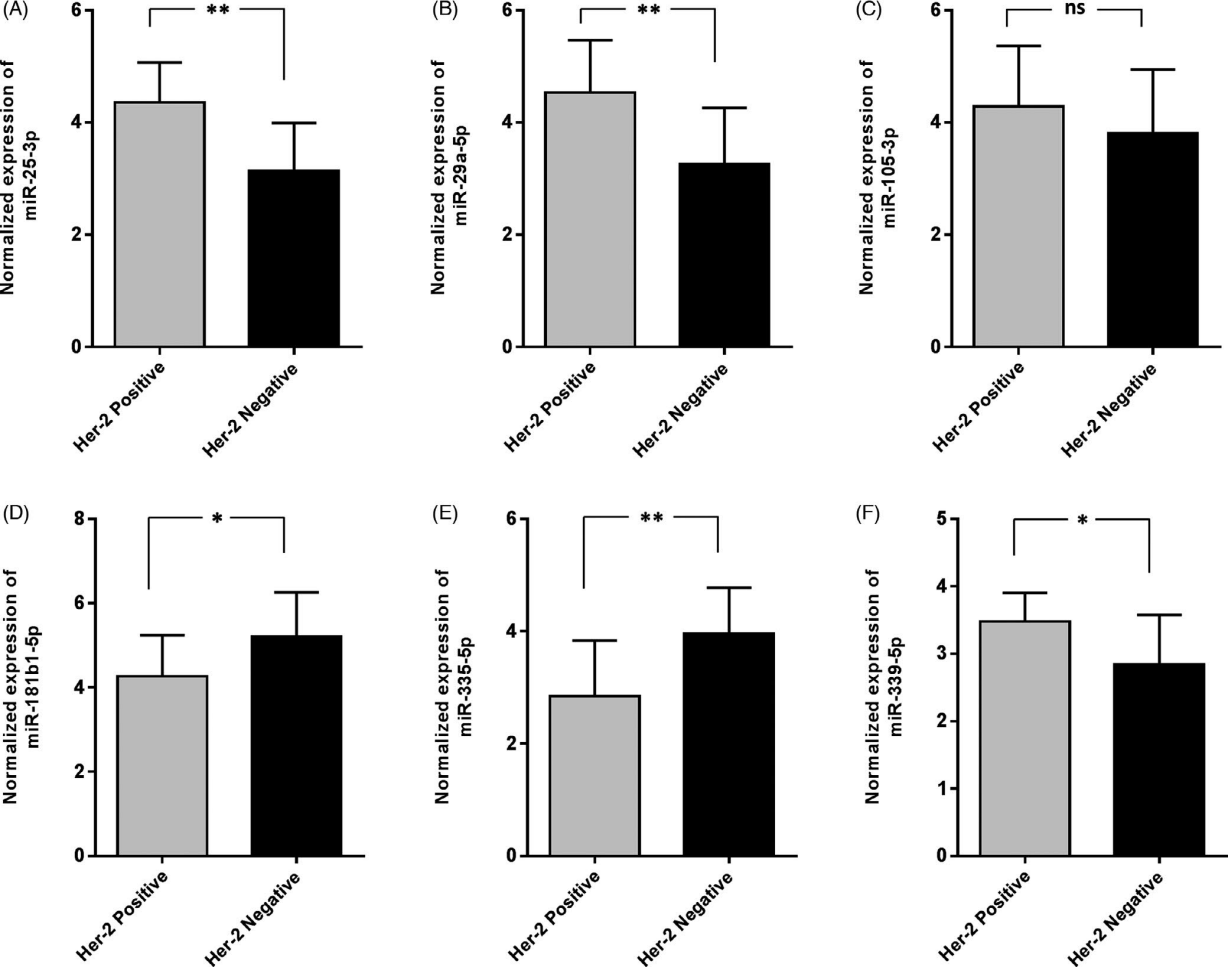
The association of the candidate miRNA expression level and HER‐2 status. a higher expression of hsa‐miR‐25‐3p, hsa‐miR‐29a‐5p, and hsa‐miR‐339‐5p was detected in patients who were HER‐2 positive. Regarding hsa‐miR‐105‐3p, a statistically insignificant higher expression of this miRNA was detected in HER‐2 positive patients. Interestingly, hsa‐miR‐181b1‐5p and hsa‐miR‐335‐5p, on the other hand, were upregulated in HER‐2 negative samples. In this figure: *p‐value <0.05, **p‐value <0.01

We also conducted an investigation into the possible association of the miRNA expression levels with HER‐2 status in BC tissues. We showed that the expression of hsa‐miR‐25‐3p, ‐29a‐5p, and ‐339‐5p was increased in HER‐2 positive samples. On the other hand, it seemed that the expression of hsa‐miR‐181b1‐5p and ‐335‐5p decreased in HER‐2 positive samples, compared to the negative specimens. Although the expression of hsa‐miR‐105‐5p increased in HER‐2 positive samples, these changes were statistically insignificant (Figure 3A–F).

### 3.3 Estimation of miRNA biomarker potentiality in BC diagnosis

**FIGURE 4:**
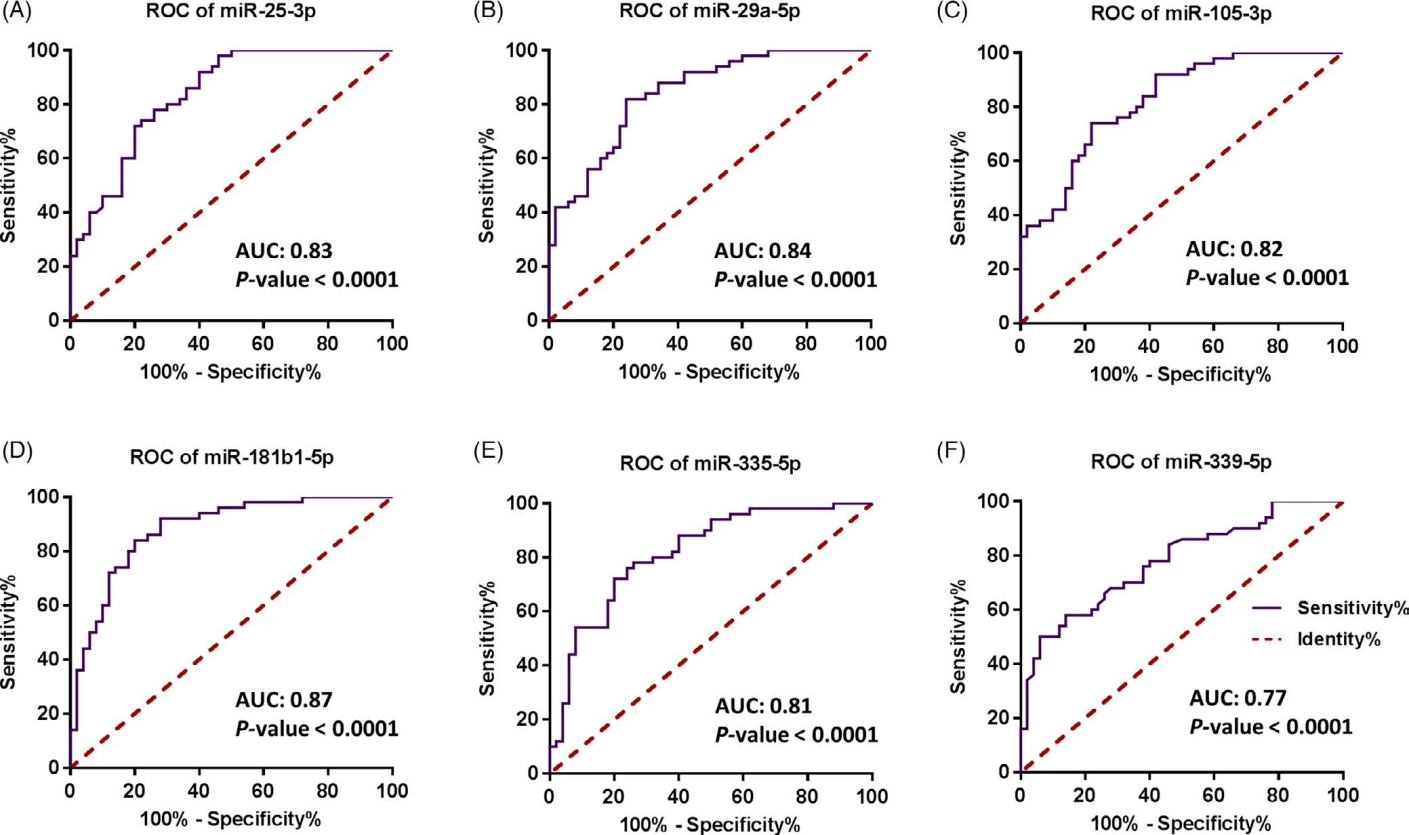
ROC curve analysis of the candidate miRNAs. The vertical axis shows sensitivity, while the horizontal one delineates specificity. The area under the curve (AUC) with at least 95% of confidence intervals (CI) was used as the main parameter to calculate whether the candidate miRNAs can be deemed as a diagnostic biomarker or not. miRNAs with AUC >0.5 were considered to function as a biomarker in BC diagnosis. The calculated AUC for the candidate miRNAs including hsa‐miR‐25‐3p (A), ‐29a‐5p (B), 105‐3p (C), ‐181b1‐5p (D), ‐335‐5p (E), and ‐339‐5p (F) was 0.83, 0.84, 0.82, 0.87, 0.81, and 0.77 (all *p*‐values <0.0001; 95% of CI), respectively

The AUC‐ROC curve was used to assess the sensitivity and specificity of each candidate miRNA analyzed for each candidate miRNA and it indicated that each miRNA could potentially be used as a tumor and/or diagnostic biomarker for BC. MiRNAs with AUC >0.50 serve as a biomarker in BC diagnosis. The calculated AUC for hsa‐miR‐25‐3p, −29a‐5p, 105‐3p, ‐181b1‐5p, ‐335‐5p, and ‐339‐5p was 0.83, 0.84, 0.82, 0.87, 0.81, and 0.77 (all *p*‐values <0.0001; 95% of CI), respectively (Figure 4A‐F).

### 3.4 Functional enrichment analysis

**FIGURE 5:**
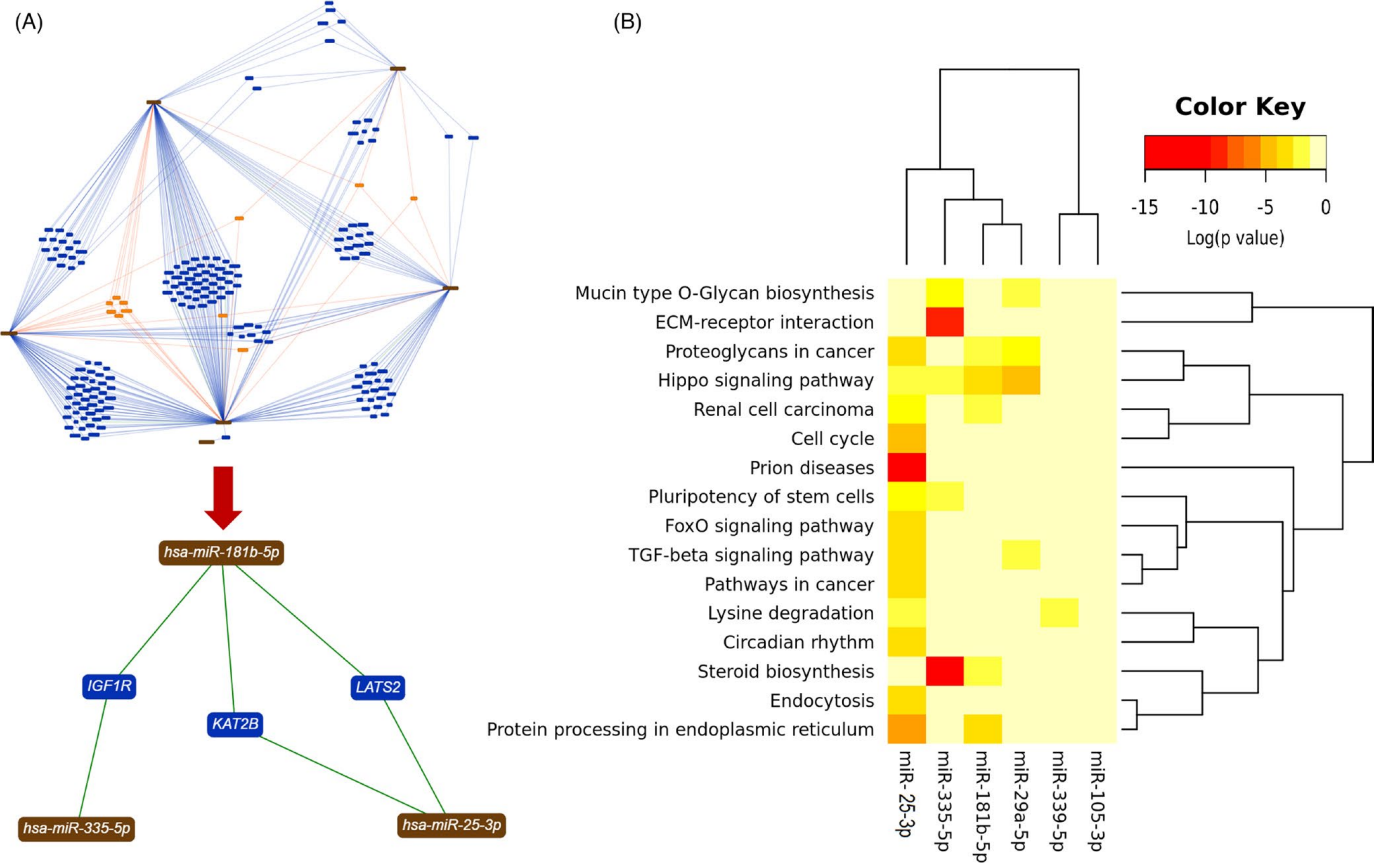
Functional enrichment analysis using in silico databases. (A) The detailed information on miRNA‐mRNA interactions in *Homo sapiens* in the form of interactive interaction networks was obtained using miRTargetLink Human. By selecting the ‘*Strong Experimental Evidence*’ option, only three miRNAs had been identified to possibly target specific pathways. These data were confirmed using miRWalk 2.0 (Figure S2). A high‐resolution figure of interaction networks is accessible in Figure S3. (B) A miRNA vs. GOSlim categories heatmap made by DIANA‐miRPath v3.0. The heatmap depicts the level of enrichment in GO categories of different miRNA family members in *Homo sapiens*

To show an integrated network for the candidate miRNAs and their potential targets, miRTargetLink Human was used (Figure S2). We only focused on the ‘*Strong Experimental Evidence*’ option and as a result, among all identified targets, *IGF1R*, *KAT2B*, and *LATS2* were identified as the common targets among hsa‐miR‐335‐5p, hsa‐miR‐25‐3p, and hsa‐miR‐181b‐5p^24^ (Figure 5A). Using the ‘*Gene and Pathway Union Analysis*’ option of DIANA‐miRPath v.3.0, we also demonstrated that the candidate miRNAs may contribute to different cell signaling such as hippo and TGF‐β signaling pathways, endocytosis, and steroid biosynthesis in cancer. Further information is accessible in Figure 5B. We also used the miRWalk 2.0 platform which in turn showed the possible interactions among the candidate miRNAs and their targets (Figure S3).

### 3.5 Survival analysis of the signature

Using the Kaplan–Meier plotter database, we determined that the overall survival BC is low in cases with the aberrant expression of each candidate miRNA. The follow‐up time was determined at a maximum of 300 months (Figure S4).

## 4 DISCUSSION

Breast cancer is the most common cancer in females worldwide^2^ and despite major advances in early diagnosis, the discovery of more reliable diagnostic and prognostic biomarkers and innovative therapeutic strategies remain the primary goal. It has been suggested that miRNAs are reliable biomarkers that can be used for diagnostic and prognostic purposes^12^; the aberrant miRNA expression reflects the status of tumor progression and drug resistance in a variety of cancers, including BC.^25^, ^26^ Different molecular miRNA signatures can also successfully discriminate BC subtypes^27^; thus, we investigated whether a signature of six candidate miRNAs has the potential to be a novel diagnostic/prognostic biomarker. To this end, the expression of each miRNA was determined using RT‐qPCR. Our findings showed that the expression of hsa‐miR‐25‐3p, ‐29a‐5p, 105‐3p, and ‐118b1‐5p was increased in BC tumors compared to the ANT tissue samples. The expression of hsa‐miR‐335‐5p and ‐339‐5p, on the other hand, was decreased in BC tumor tissues. There was a correlation between the expression levels of the miRNAs and certain BC clinicopathological characteristics. These findings indicate that the signature of six miRNAs is a reliable biomarker for early detection of BC.

Hsa‐miR‐25‐3p promotes tumor cell proliferation by targeting tumor suppressor *BTG2*; this miRNA is a new diagnostic and therapeutic target in triple‐negative BC^28^; however, its potential to be a reliable biomarker in BC is still unclear. Likewise, exosomal miR‐25‐3p is involved in pre‐metastatic niche formation of colorectal cancer and is used as a biomarker.^29^ Herein, we showed that the expression of this miRNA was increased in tumor tissues, especially in the advanced TNM stages and in HER‐2 positive patients. MiR‐25 cuts both ways because it plays as an oncogenic miRNA in esophageal cancer,^30^ cholangiocarcinoma,^31^ gastric cancer,^11^ and lung cancer,^32^ while it is a tumor suppressor in colon cancer^33^ and anaplastic thyroid carcinoma.^34^ Herein, we showed that hsa‐miR25‐3p plays an oncogenic role in BC progression, and ROC curve analysis also showed that this miRNA can be used as a biomarker for early diagnosis of BC; *in silico* analysis demonstrated that this miRNA is dysregulated in BC patients with poor prognosis; however, further studies are needed to unveil the exact molecular mechanisms whereby this miRNA functions in BC development.

Hsa‐miR‐29a‐5p has been detected to play fundamentally in BC development and invasion.^35^ Upregulation of this miRNA was also reported in BC serum and tissue samples.^36^ MiR‐29a inhibits tristetraprolin expression and thus controls BC cell epithelial–mesenchymal transition and metastasis.^37^ Given such findings, we hypothesized whether hsa‐miR‐29a‐5p is a BC biomarker; our findings showed that the expression of this miRNA increased in tumor samples (especially in advanced TNM stages, videlicet stage III) and also in HER‐2 positive cells. This may underscore the oncogenic roles of miR‐29a in BC, confirmed by the previous investigations attributed such roles to BC development.^35^ On the other hand, Wu et al. showed the inhibitory roles of miR‐29a‐5p that can suppress tumor growth by down‐regulating B‐Myb.^38^ ROC curve and *in silico* analyses suggested that miR‐29a is a diagnostic and prognostic biomarker. These contradictory roles of hsa‐miR‐29a‐5p in BC development and the underlying molecular mechanisms need to be unearthed.

As an oncogene, hsa‐miR‐105‐3p promotes the proliferation and metastasis of BC cells by targeting Golgi integral membrane protein 4.^39^ This finding was in line with our data showing the increased expression of this miRNA in tumor samples. This miRNA is associated with the occurrence and development of different cancers such as ovarian cancer,^40^ prostate cancer,^41^ colon cancer,^42^ and hepatocellular carcinoma.^43^ MiR‐105 was substantially upregulated in esophageal cancer tissues and its overexpression was significantly correlated with positive lymph node metastasis, advanced TNM stage, and poor overall survival.^44^ We also found that the expression of this miRNA is associated with the advanced TNM stages. Li et al. showed that the combined circulating miR‐105/93‐3p levels are a diagnostic biomarker for early and advanced stages of triple‐negative BC.^27^ In sum, we showed that hsa‐miR‐105‐3p is a diagnostic biomarker in BC; additionally, *in silico* predictions attributed prognostic values for this miRNA.

Herein, we showed that the hsa‐miR‐181b1‐5p was upregulated in tumor samples in comparison with ANT tissues. This upregulation was also correlated with advanced TNM stages and HER‐2 negative status in BC patients. Different studies *in vitro* and *in vivo* demonstrated the upregulation of miR‐181b1‐5p in highly metastatic BC cell lines.^45^, ^46^, ^47^, ^48^ Overexpression of miR‐181b induces breast tumorigenesis and aggressiveness.^45^ It suppresses the expression of the proapoptotic Bim signal, stimulating cell‐cycle dysregulation, overgrowth, and tumorigenesis.^49^, ^50^ Despite its different roles, the potential application of miR‐181b1 as a BC biomarker yet still is blanketed in mystery. Herein, we showed that hsa‐miR‐181b1‐5p is a reliable biomarker for early diagnosis of BC; furthermore, the patients with dysregulated miR‐181b1 showed a poor prognosis.

MiR‐335 suppresses metastasis in BC cells, i.e., its decreased expression develops BC metastasis in mice.^51^ Downregulation of miR‐335 is associated with tumor aggressiveness and a poor prognosis.^52^ MiR‐335 suppresses BC cell migration by negatively regulating the HGF/c‐Met pathway.^53^ Liu et al. showed the downregulation of miR‐335‐5p in BC^54^; in line with this finding, our data underscored the tumor‐suppressive role of this miRNA in BC. These were consistent with some studies, showing that miR‐335 inhibits the proliferation, migration, and invasion of BC cells by targeting *erythropoietin‐producing hepatocellular A4*.^55^ We also demonstrated that the expression of hsa‐miR‐335‐5p increased in the initial stages of tumorigenesis (stages I/II), and was reversely correlated with positive HER‐2 status. The AUC‐ROC curve analysis suggested that this miRNA is a reliable biomarker with good sensitivity and specificity. Moreover, aberrant expression of this miRNA was seen in patients with a poor prognosis.

Using *in silico* available tools, we also showed that hsa‐miR‐335‐5p, hsa‐miR‐25‐3p, and hsa‐miR‐181b‐5p target *IGF1R*, *KAT2B*, and *LATS2* in common. Different studies have shown that up to 50% of breast tumors express the activated form of IGF1R. IGF1R bestows the stem cell characteristics and therapy resistance to breast tumors.^56^ FOXP3‐KAT2B axis plays important role in BC metastasis.^57^ LATS2 axis regulates the cell cycle and its dysregulation is associated with cell growth in BC.^58^

Hsa‐miR‐339‐5p is a tumor suppressor in different cancers such as BC,^59^ hepatocellular carcinoma,^60^ ovarian cancer,^61^ colorectal cancer,^62^ and melanoma.^63^ This miRNA has been identified to substantially decrease BC cell migration and invasion capacity.^59^ Hsa‐miR‐339‐5p is associated with the poor prognosis in BC patients, a process that is imputed to the regulation by long non‐coding RNA MALAT1 and MAFG‐AS1.^64^, ^65^ Our study verified the tumor‐suppressive and diagnostic biomarker functions of this miRNA in BC samples.

This study has several limitations: to begin with, we did not check the expression levels of each candidate miRNA in serum or plasma levels; further studies are needed to check the exosomal miRNAs in serum or plasma samples of BC patients. The possibility of a serological test that can augment histological information of a tumor without the need for biopsy is an exciting approach for research and clinical application. Secondly, miRNA expression profiling is required to show which genes are regulated by the signature. Thirdly, larger studies with more diverse samples would be helpful for confirming our data.

In this study, we demonstrated that a signature of six miRNAs (i.e., hsa‐miR‐25‐3p, ‐29a‐5p, 105‐3p, ‐181b1‐5p, ‐335‐5p, and ‐339‐5p) effectively distinguishes the tumor and ANT tissues with acceptable sensitivity and specificity; however, further steps should be taken forward to show the underlying molecular mechanisms whereby such miRNAs function in BC development.

## CONFLICT OF INTEREST

The authors declare no conflict of interest.

## Supporting information

App S1

## References

[1] Park EH , Min SY , Kim Z , et al. Basic facts of breast cancer in Korea in 2014: the 10‐year overall survival progress. J Breast Cancer. 2017;20(1):1. 28382089 10.4048/jbc.2017.20.1.1 PMC5378568

[2] Bitaraf A , Babashah S , Garshasbi M . Aberrant expression of a five‐microRNA signature in breast carcinoma as a promising biomarker for diagnosis. J Clin Lab Anal. 2020;34(2):e23063. 31595567 10.1002/jcla.23063 PMC7031575

[3] Tahmouresi F , Razmara E , Pakravan K , et al. Upregulation of the long noncoding RNAs DSCAM‐AS1 and MANCR is a potential diagnostic marker for breast carcinoma. Biotechnol Appl Biochem. 2020;67(5):1‐13. 10.1002/bab.2048 33012018

[4] Ghaffari‐Makhmalbaf P , Sayyad M , Pakravan K , et al. Docosahexaenoic acid reverses the promoting effects of breast tumor cell‐derived exosomes on endothelial cell migration and angiogenesis. Life Sci. 2021;264:118719. 33159957 10.1016/j.lfs.2020.118719

[5] Schulz‐Wendtland R , Fuchsjäger M , Wacker T , Hermann K‐P . Digital mammography: an update. Eur J Radiol. 2009;72(2):258‐265. 19592186 10.1016/j.ejrad.2009.05.052

[6] Mori M , Akashi‐Tanaka S , Suzuki S , et al. Diagnostic accuracy of contrast‐enhanced spectral mammography in comparison to conventional full‐field digital mammography in a population of women with dense breasts. Breast Cancer. 2017;24(1):104‐110. 26942415 10.1007/s12282-016-0681-8

[7] von Euler‐Chelpin M , Lillholm M , Vejborg I , Nielsen M , Lynge E . Sensitivity of screening mammography by density and texture: a cohort study from a population‐based screening program in Denmark. Breast Cancer Res. 2019;21(1):1‐7. 31623646 10.1186/s13058-019-1203-3 PMC6796411

[8] Lagerlund M , Sontrop JM , Zackrisson S . Psychosocial factors and attendance at a population‐based mammography screening program in a cohort of Swedish women. BMC Womens Health. 2014;14(1):1‐9. 24565263 10.1186/1472-6874-14-33 PMC3942217

[9] Bitaraf A , Razmara E , Bakhshinejad B , et al. The oncogenic and tumor suppressive roles of RNA‐binding proteins in human cancers. J Cell Physiol. 2021;236(9):6200‐6224. 33559213 10.1002/jcp.30311

[10] Maminezhad H , Ghanadian S , Pakravan K , et al. A panel of six‐circulating miRNA signature in serum and its potential diagnostic value in colorectal cancer. Life Sci. 2020;258:118226. 32771555 10.1016/j.lfs.2020.118226

[11] Poursheikhani A , Bahmanpour Z , Razmara E , et al. Non‐coding RNAs underlying chemoresistance in gastric cancer. Cell Oncol. 2020;43:1‐28. 10.1007/s13402-020-00528-2 PMC12990740 32495294

[12] Razmara E , Bitaraf A , Yousefi H , et al. Non‐coding RNAs in cartilage development: an updated review. Int J Mol Sci. 2019;20(18):4475. 10.3390/ijms20184475 PMC6769748 31514268

[13] Hussen BM , Hidayat HJ , Salihi A , Sabir DK , Taheri M , Ghafouri‐Fard S . MicroRNA: a signature for cancer progression. Biomed Pharmacother. 2021;138:111528. 33770669 10.1016/j.biopha.2021.111528

[14] Babashah S , Soleimani M . The oncogenic and tumour suppressive roles of microRNAs in cancer and apoptosis. Eur J Cancer. 2011;47(8):1127‐1137. 21402473 10.1016/j.ejca.2011.02.008

[15] Razmara E , Salehi M , Aslani S , et al. Graves' disease: introducing new genetic and epigenetic contributors. J Mol Endocrinol. 2021;66(2):R33‐R55. 33295879 10.1530/JME-20-0078

[16] Ghafouri‐Fard S , Hussen BM , Badrlou E , Abak A , Taheri M . MicroRNAs as important contributors in the pathogenesis of colorectal cancer. Biomed Pharmacother. 2021;140:111759. 34091180 10.1016/j.biopha.2021.111759

[17] Azzopardi J , Chepick O , Hartmann W , et al. The World Health Organization histological typing of breast tumors—Second edition. Am J Clin Pathol. 1982;78(6):806‐816. 7148748 10.1093/ajcp/78.6.806

[18] Vlachos IS , Zagganas K , Paraskevopoulou MD , et al. DIANA‐miRPath v3. 0: deciphering microRNA function with experimental support. Nucleic Acids Res. 2015;43(W1):W460‐W466. 25977294 10.1093/nar/gkv403 PMC4489228

[19] Kern F , Aparicio‐Puerta E , Li Y , et al. miRTargetLink 2.0—interactive miRNA target gene and target pathway networks. Nucleic Acids Res. 2021;49:W409‐W416. 34009375 10.1093/nar/gkab297 PMC8262750

[20] Sticht C , De La Torre C , Parveen A , Gretz N . miRWalk: an online resource for prediction of microRNA binding sites. PLoS ONE. 2018;13(10):e0206239. 30335862 10.1371/journal.pone.0206239 PMC6193719

[21] Pal SK , Hurria A . Impact of age, sex, and comorbidity on cancer therapy and disease progression. J Clin Oncol. 2010;28(26):4086‐4093. 20644100 10.1200/JCO.2009.27.0579

[22] Györffy B , Lanczky A , Eklund AC , et al. An online survival analysis tool to rapidly assess the effect of 22,277 genes on breast cancer prognosis using microarray data of 1,809 patients. Breast Cancer Res Treat. 2010;123(3):725‐731. 20020197 10.1007/s10549-009-0674-9

[23] Weinstein JN , Collisson EA , Mills GB , et al. The cancer genome atlas pan‐cancer analysis project. Nat Genet. 2013;45(10):1113‐1120. 24071849 10.1038/ng.2764 PMC3919969

[24] George ML , Tutton MG , Janssen F , et al. Vegf‐a, vegf‐c, and vegf‐d in colorectal cancer progression. Neoplasia. 2001;3(5):420. 11687953 10.1038/sj.neo.7900186 PMC1506210

[25] Robertson NM , Yigit MV . The role of microRNA in resistance to breast cancer therapy. Wiley Interdiscip Rev. 2014;5(6):823‐833. 10.1002/wrna.1248 25044299

[26] Negrini M , Calin GA . Breast cancer metastasis: a microRNA story. Breast Cancer Res. 2008;10(2):1‐4. 10.1186/bcr1867 PMC2397516 18373886

[27] Li H‐Y , Liang J‐L , Kuo Y‐L , et al. miR‐105/93‐3p promotes chemoresistance and circulating miR‐105/93‐3p acts as a diagnostic biomarker for triple negative breast cancer. Breast Cancer Res. 2017;19(1):1‐14. 29258605 10.1186/s13058-017-0918-2 PMC5738224

[28] Chen H , Pan H , Qian Y , Zhou W , Liu X . MiR‐25‐3p promotes the proliferation of triple negative breast cancer by targeting BTG2. Mol Cancer. 2018;17(1):1‐11. 29310680 10.1186/s12943-017-0754-0 PMC5759260

[29] Zeng Z , Li Y , Pan Y , et al. Cancer‐derived exosomal miR‐25‐3p promotes pre‐metastatic niche formation by inducing vascular permeability and angiogenesis. Nat Commun. 2018;9(1):1‐14. 30568162 10.1038/s41467-018-07810-w PMC6300604

[30] Wu C , Li M , Hu C , Duan H . Clinical significance of serum miR‐223, miR‐25 and miR‐375 in patients with esophageal squamous cell carcinoma. Mol Biol Rep. 2014;41(3):1257‐1266. 24390317 10.1007/s11033-013-2970-z

[31] Liu H , Ma L , Wang J . Overexpression of miR‐25 is associated with progression and poor prognosis of cholangiocarcinoma. Exp Ther Med. 2019;18(4):2687‐2694. 31555370 10.3892/etm.2019.7844 PMC6755412

[32] Wu T , Chen W , Kong D , et al. miR‐25 targets the modulator of apoptosis 1 gene in lung cancer. Carcinogenesis. 2015;36(8):925‐935. 25998847 10.1093/carcin/bgv068

[33] Li Q , Zou C , Zou C , et al. MicroRNA‐25 functions as a potential tumor suppressor in colon cancer by targeting Smad7. Cancer Lett. 2013;335(1):168‐174. 23435373 10.1016/j.canlet.2013.02.029

[34] Esposito F , Tornincasa M , Pallante P , et al. Down‐regulation of the miR‐25 and miR‐30d contributes to the development of anaplastic thyroid carcinoma targeting the polycomb protein EZH2. J Clin Endocrinol. 2012;97(5):E710‐E718. 10.1210/jc.2011-3068 22399519

[35] Li Z‐H , Xiong Q‐Y , Xu L , et al. miR‐29a regulated ER‐positive breast cancer cell growth and invasion and is involved in the insulin signaling pathway. Oncotarget. 2017;8(20):32566. 28427228 10.18632/oncotarget.15928 PMC5464809

[36] Raeisi F , Mahmoudi E , Dehghani‐Samani M , et al. Differential expression profile of miR‐27b, miR‐29a, and miR‐155 in chronic lymphocytic leukemia and breast cancer patients. Mol Ther Oncolytics. 2020;16:230‐237. 32123723 10.1016/j.omto.2020.01.004 PMC7037977

[37] Gebeshuber CA , Zatloukal K , Martinez J . miR‐29a suppresses tristetraprolin, which is a regulator of epithelial polarity and metastasis. EMBO Rep. 2009;10(4):400‐405. 19247375 10.1038/embor.2009.9 PMC2672883

[38] Wu Z , Huang X , Huang X , Zou Q , Guo Y . The inhibitory role of Mir‐29 in growth of breast cancer cells. J Exp Clin Cancer Res. 2013;32(1):1‐7. 24289849 10.1186/1756-9966-32-98 PMC4176287

[39] Lin B , Liu C , Shi E , et al. MiR‐105‐3p acts as an oncogene to promote the proliferation and metastasis of breast cancer cells by targeting GOLIM4. BMC Cancer. 2021;21(1):1‐10. 33722196 10.1186/s12885-021-07909-2 PMC7962220

[40] Li M , Zhang S , Ma Y , Yang Y , An R . Role of hsa‐miR‐105 during the pathogenesis of paclitaxel resistance and its clinical implication in ovarian cancer. Oncol Rep. 2021;45(5):1‐13. 33846814 10.3892/or.2021.8035 PMC8025119

[41] Honeywell DR , Cabrita MA , Zhao H , Dimitroulakos J , Addison CL . miR‐105 inhibits prostate tumour growth by suppressing CDK6 levels. PLoS ONE. 2013;8(8):e70515. 23950948 10.1371/journal.pone.0070515 PMC3737265

[42] Wang Z , Zhang J , Yang B , et al. Long intergenic noncoding RNA 00261 acts as a tumor suppressor in non‐small cell lung cancer via regulating miR‐105/FHL1 axis. J Cancer. 2019;10(25):6414. 31772674 10.7150/jca.32251 PMC6856729

[43] Ma Y‐S , Wu T‐M , Lv Z‐W , et al. High expression of miR‐105‐1 positively correlates with clinical prognosis of hepatocellular carcinoma by targeting oncogene NCOA1. Oncotarget. 2017;8(7):11896. 28060733 10.18632/oncotarget.14435 PMC5355313

[44] Gao R , Wang Z , Liu Q , Yang C . MicroRNA‐105 plays an independent prognostic role in esophageal cancer and acts as an oncogene. Cancer Biomark. 2020;27(2):173‐180.3179666310.3233/CBM-180

[45] Taha M , Mitwally N , Soliman AS , Yousef E . Potential diagnostic and prognostic utility of miR‐141, miR‐181b1, and miR‐23b in breast cancer. Int J Mol Sci. 2020;21(22):8589. 10.3390/ijms21228589 PMC7697480 33202602

[46] Menschikowski M , Hagelgans A , Nacke B , Jandeck C , Sukocheva O , Siegert G . Epigenetic control of phospholipase A 2 receptor expression in mammary cancer cells. BMC Cancer. 2015;15(1):1‐9. 26672991 10.1186/s12885-015-1937-y PMC4682251

[47] Chang Y‐Y , Kuo W‐H , Hung J‐H , et al. Deregulated microRNAs in triple‐negative breast cancer revealed by deep sequencing. Mol Cancer. 2015;14(1):1‐13. 25888956 10.1186/s12943-015-0301-9 PMC4351690

[48] Yan L‐X , Huang X‐F , Shao Q , et al. MicroRNA miR‐21 overexpression in human breast cancer is associated with advanced clinical stage, lymph node metastasis and patient poor prognosis. RNA. 2008;14(11):2348‐2360. 18812439 10.1261/rna.1034808 PMC2578865

[49] Mansueto G , Forzati F , Ferraro A , et al. Identification of a new pathway for tumor progression: MicroRNA‐181b up‐regulation and CBX7 down‐regulation by HMGA1 protein. Genes Cancer. 2010;1(3):210‐224. 21779448 10.1177/1947601910366860 PMC3092193

[50] D'Ippolito E , Iorio MV . MicroRNAs and triple negative breast cancer. Int J Mol Sci. 2013;14(11):22202‐22220. 24284394 10.3390/ijms141122202 PMC3856060

[51] Tavazoie SF , Alarcón C , Oskarsson T , et al. Endogenous human microRNAs that suppress breast cancer metastasis. Nature. 2008;451(7175):147‐152. 18185580 10.1038/nature06487 PMC2782491

[52] Heyn H , Engelmann M , Schreek S , et al. MicroRNA miR‐335 is crucial for the BRCA1 regulatory cascade in breast cancer development. Int J Cancer. 2011;129(12):2797‐2806. 21618216 10.1002/ijc.25962

[53] Gao Y , Zeng F , Wu J‐Y , et al. MiR‐335 inhibits migration of breast cancer cells through targeting oncoprotein c‐Met. Tumor biology. 2015;36(4):2875‐2883. 25492484 10.1007/s13277-014-2917-6

[54] Liu J , Mao Q , Liu Y , Hao X , Zhang S , Zhang J . Analysis of miR‐205 and miR‐155 expression in the blood of breast cancer patients. Chin J Cancer Res. 2013;25(1):46. 23372341 10.3978/j.issn.1000-9604.2012.11.04 PMC3555294

[55] Dong Y , Liu Y , Jiang A , Li R , Yin M , Wang Y . MicroRNA‐335 suppresses the proliferation, migration, and invasion of breast cancer cells by targeting EphA4. Mol Cell Biochem. 2018;439(1):95‐104. 28795314 10.1007/s11010-017-3139-1

[56] Farabaugh SM , Boone DN , Lee AV . Role of IGF1R in breast cancer subtypes, stemness, and lineage differentiation. Front Endocrinol (Lausanne). 2015;6:59. 25964777 10.3389/fendo.2015.00059 PMC4408912

[57] Zhang G , Zhang W , Li B , et al. MicroRNA‐200c and microRNA‐141 are regulated by a FOXP3‐KAT2B axis and associated with tumor metastasis in breast cancer. Breast Cancer Res. 2017;19(1):1‐13. 28637482 10.1186/s13058-017-0858-x PMC5480201

[58] Hua K , Jin J , Zhao J , et al. miR‐135b, upregulated in breast cancer, promotes cell growth and disrupts the cell cycle by regulating LATS2. Int J Oncol. 2016;48(5):1997‐2006. 26934863 10.3892/ijo.2016.3405

[59] Wu Z‐S , Wu Q , Wang C‐Q , et al. MiR‐339‐5p inhibits breast cancer cell migration and invasion in vitro and may be a potential biomarker for breast cancer prognosis. BMC Cancer. 2010;10(1):1‐10. 20932331 10.1186/1471-2407-10-542 PMC2958952

[60] Wang Y‐L , Chen C‐M , Wang X‐M , Wang L . Effects of miR‐339‐5p on invasion and prognosis of hepatocellular carcinoma. Clin Res Hepatol Gastroenterol. 2016;40(1):51‐56. 26186881 10.1016/j.clinre.2015.05.022

[61] Shan W , Li J , Bai Y , Lu X . miR‐339‐5p inhibits migration and invasion in ovarian cancer cell lines by targeting NACC1 and BCL6. Tumor Biology. 2016;37(4):5203‐5211. 26553360 10.1007/s13277-015-4390-2

[62] Zhou C , Liu G , Wang L , et al. MiR‐339‐5p regulates the growth, colony formation and metastasis of colorectal cancer cells by targeting PRL‐1. PLoS ONE. 2013;8(5):e63142. 23696794 10.1371/journal.pone.0063142 PMC3656035

[63] Weber CE , Luo C , Hotz‐Wagenblatt A , et al. miR‐339‐3p is a tumor suppressor in melanoma. Cancer Res. 2016;76(12):3562‐3571. 27197185 10.1158/0008-5472.CAN-15-2932

[64] Zheng L , Zhang Y , Fu Y , et al. Long non‐coding RNA MALAT1 regulates BLCAP mRNA expression through binding to miR‐339‐5p and promotes poor prognosis in breast cancer. Biosci Rep. 2019;39(2):BSR20181284. 30683807 10.1042/BSR20181284 PMC6379223

[65] Li H , Zhang G , Pan C , Zhang X , Su X . LncRNA MAFG‐AS1 promotes the aggressiveness of breast carcinoma through regulating miR‐339‐5p/MMP15. Eur Rev Med Pharmacol Sci. 2019;23(7):2838‐2846. 31002134 10.26355/eurrev_201904_17561

