# Supplementary material for: Identification of a six‐microRNA signature as a potential diagnostic biomarker in breast cancer tissues
Source: J Clin Lab Anal. 2021 Sep 15;35(11):e24010. doi: 10.1002/jcla.24010 (PMC8605139; doi:10.1002/jcla.24010)
Supplement: Supplementary file 1 — App S1 [file JCLA-35-e24010-s001.docx]

**Supplementary Data**

**Supplementary Table S1**. Slope and efficiencies of standard curves for each primer set


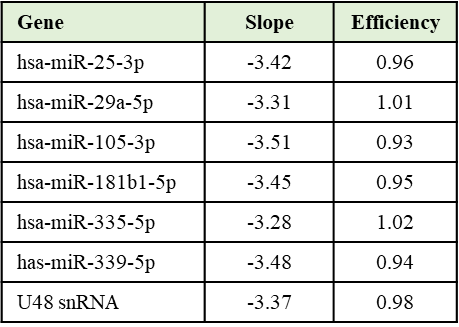


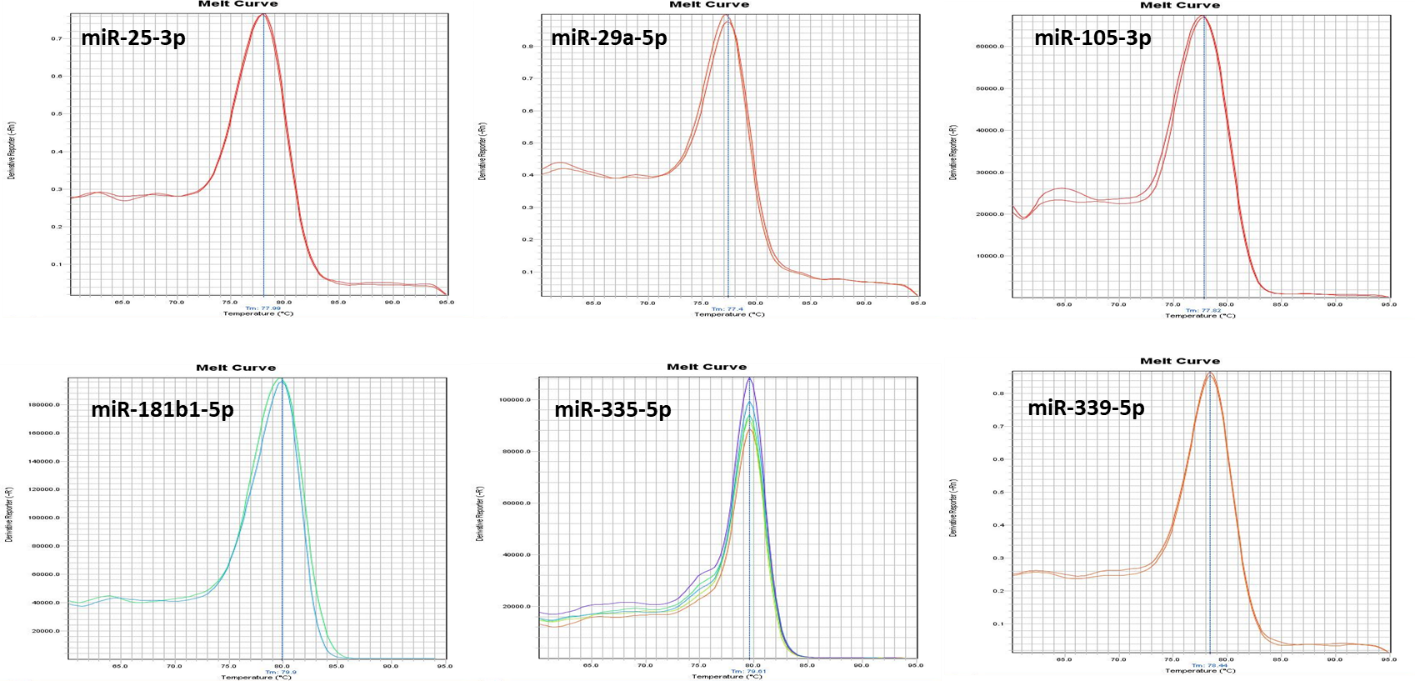


**Supplementary Figure 1: Uniqueness and specificity assessment of the RT-qPCR products through melting curve analysis.** Melting curve analysis performed on PCR products obtained from amplification reactions for miR-25-3p, miR-29a-5p, miR-105-3p, miR-181b1-5p, miR-335-5p, and miR-339-5p. The curves featured by a single and sharp peak at expected Tm.


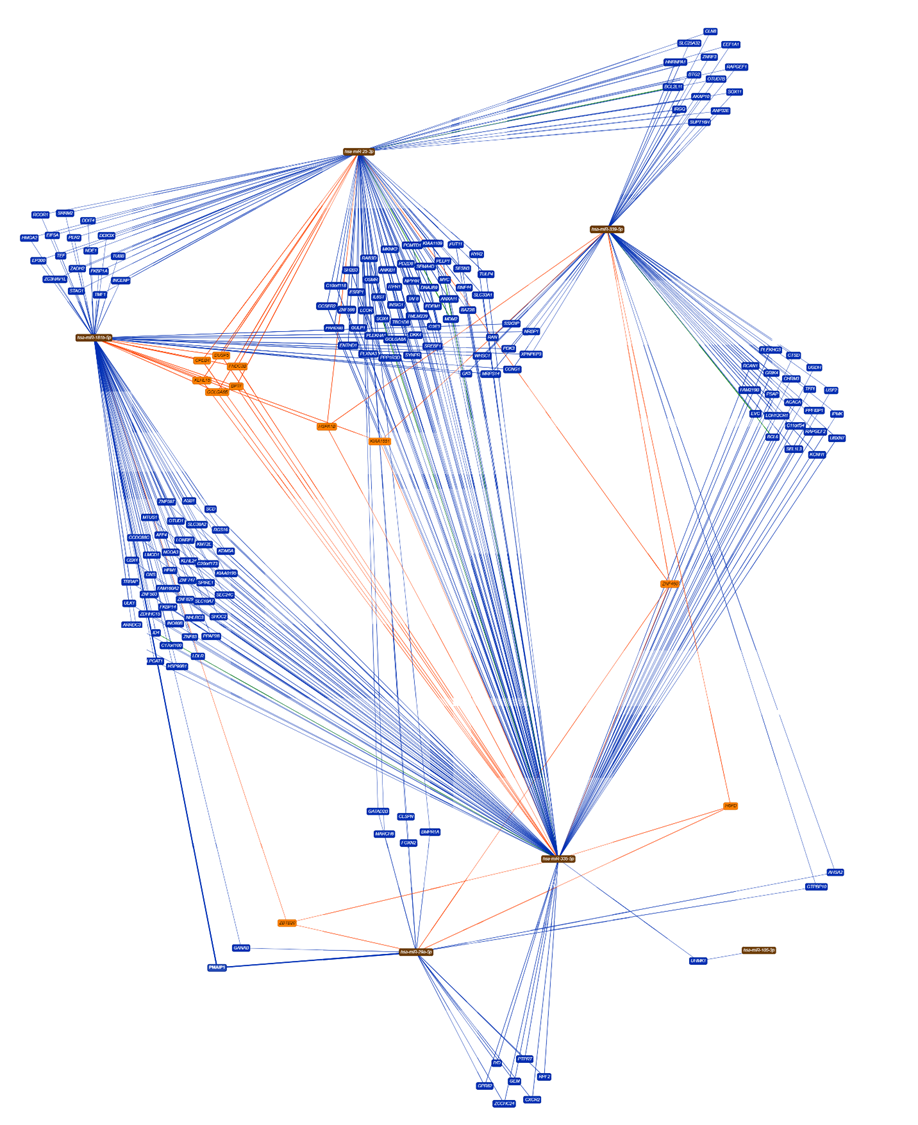


**Supplementary Figure 2.** A high-resolution figure of interaction networks created by miRTargetLink Human.

**
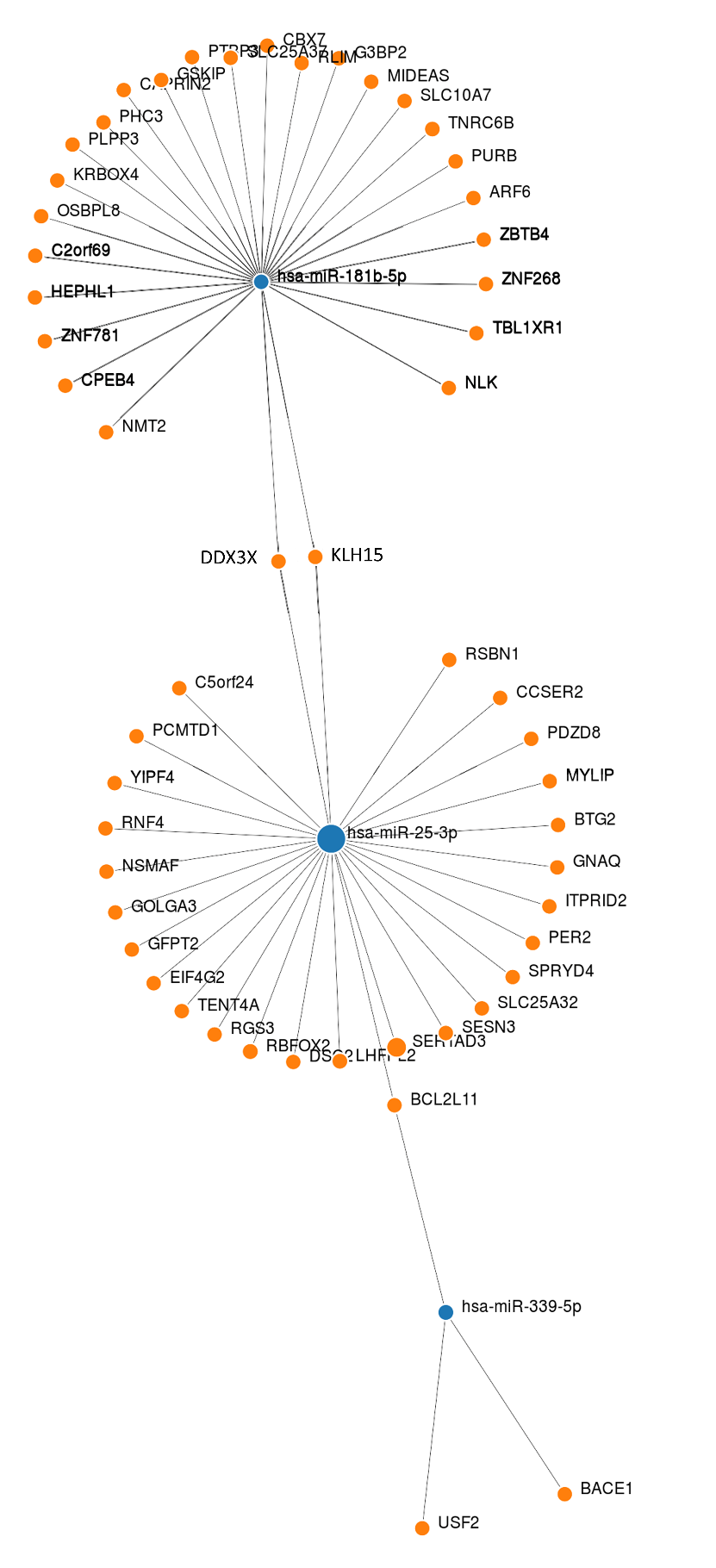
**

**Supplementary Figure 3.** Target Mining was performed for each miRNA using the miRWalk 2.0 online platform.


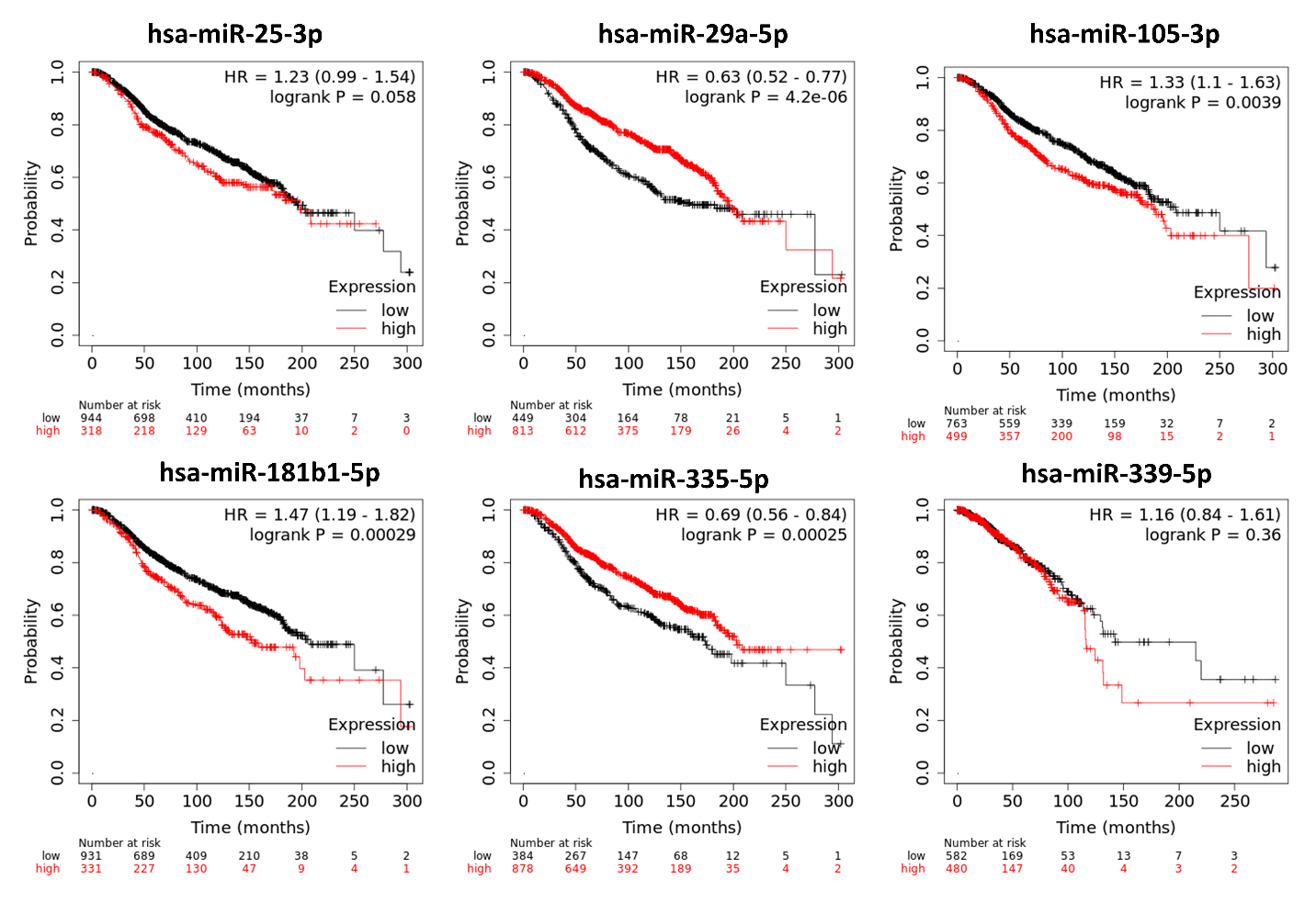


**Supplementary Figure 4**. The breast cancer dataset from Kaplan Meier Plotter was used to test for survival prediction capacity of each candidate miRNA. Samples were divided into Low (black) and High (red) expression groups for each target. Hazard ratio (HR) and *P*-value for each association are shown within each plot.
